# Canine leishmaniosis in Tunisia: Growing prevalence, larger zones of infection

**DOI:** 10.1371/journal.pntd.0009990

**Published:** 2021-12-10

**Authors:** Ali Bouattour, Amine Amri, Jaber Amine Belkhiria, Adel Rhim, Ons Fezaa, Jean-Charles Gantier, Youmna M’ghirbi

**Affiliations:** 1 Laboratoire de Virus, Vecteurs et Hôtes: LR20IPT02, Institut Pasteur de Tunis, Université Tunis El Manar, Tunis-Belvédère, Tunisia; 2 One Health Institute, University of California, Davis, Davis, California, United States of America; 3 Université de Reims Champagne-Ardenne, Faculté de Pharmacie, France; University of Iowa, UNITED STATES

## Abstract

**Background:**

Discovered by Nicolle and Comte in 1908 in Tunisia, *Leishmania infantum* is an intracellular protozoan responsible for zoonotic canine leishmaniosis (CanL) and zoonotic human visceral leishmaniasis (HVL). It is endemic in several regions of the world, including Tunisia, with dogs considered as the main domestic reservoir. The geographic expansion of canine leishmaniosis (CanL) has been linked to global environmental changes that have affected the density and the distribution of its sand fly vectors.

**Methodology/Principal findings:**

In this study, a cross-sectional epidemiological survey on CanL was carried out in 8 localities in 8 bioclimatic areas of Tunisia. Blood samples were taken from 317 dogs after clinical examination. Collected sera were tested by indirect fluorescent antibody test (IFAT; 1:80) for the presence of anti-*Leishmania infantum* antibodies. The overall seroprevalence was 58.3% (185/317). Among positive dogs, only 16.7% showed clinical signs suggestive of leishmaniosis. Seroprevalence rates varied from 6.8% to 84.6% and from 28% to 66% by bioclimatic zone and age group, respectively. Serological positivity was not statistically associated with gender. The presence of *Leishmania* DNA in blood, using PCR, revealed 21.2% (64/302) prevalence in dogs, which varied by bioclimatic zone (7.3% to 31%) and age group (7% to 25%). The entomological survey carried out in the studied localities showed 16 species of the two genera (*Phlebotomus* and *Sergentomyia)*. *P*. *perniciosus*, *P*. *papatasi*, and *P*. *perfiliewi* were the most dominant species with relative abundances of 34.7%, 25% and 20.4%, respectively.

**Conclusions/Significance:**

The present report suggests a significant increase of CanL in all bioclimatic areas in Tunisia and confirms the ongoing spread of the infection of dogs to the country’s arid zone. Such an expansion of infection in dog population could be attributed to ecological, agronomic, social and climatic factors that affect the presence and density of the phlebotomine vectors.

## Author summary

Visceral leishmaniasis caused by *Leishmania infantum* is an endemic disease in several regions of the world, especially, in the Mediterranean basin, where dogs constitute the main domestic reservoir for humans. This zoonotic vector-borne disease is closely associated with the environment.

We carried out studies in 8 different regions of Tunisia, collecting and testing blood samples from 317 dogs. Out of which, more than half (58.3%) tested positive for *L*. *infantum* (IFAT*+*), although, only one-third of these displayed clinical signs. However, the detection of *Leishmania* DNA by blood PCR revealed a global prevalence in dogs of 21.2% (64/302) with a variation between bioclimatic zones. Our entomological survey revealed 16 sand fly species, dominated by the *Phlebotomus* and *Sergentomyia* genera, of which the most dominant were *P*. *perniciosus*, *P*. *perfiliewi* and *P*. *papatasi*. The first two of these are recognized vectors of *L*. *infantum*. Canine leishmaniosis continues to spread across Tunisia, moving southward, to arid zones because of global warming and agronomic and social factors affecting the presence and density of the phlebotomine vectors.

## Introduction

Global environmental changes (e.g. global warming, deforestation, land use) and the effects of such changes on wildlife species and insect fauna are potentially affecting vector-borne diseases such as leishmaniasis [1–5]. Discovered by Nicolle and Comte in 1908 in Tunisia, *Leishmania infantum* is an intracellular protozoan that causes canine leishmaniosis (CanL) and Human visceral leishmaniasis (HVL). It has become endemic in the Mediterranean basin, the Middle East, South America, and Asia [6]. Its geographical expansion has been proposed to be linked to global environmental changes affecting the distribution of both the pathogen’s sand fly “vectors” (order Diptera, family Psychodidae, subfamily Phlebotominae) and also of the reservoirs of *L*. *infantum* composed from a wide range of mammal species, especially domestic and stray dogs (*Canis familiaris*) [7].

An estimated 50000 to 90000 new visceral leishmaniasis cases occur worldwide annually. Most cases occur in Brazil, East Africa and in India with primary signs that include irregular bouts of fever, weight loss, anemia and hepato-splenomegaly [6].

In Tunisia, human visceral leishmaniasis (HVL) is caused by the same *Leishmania* species zymodeme MON-1 (set of strains with the same enzymatic profile) [8–10]. The parasite is transmitted to mammalian hosts through bites of infected phlebotomine sandflies of the genus *Phlebotomus*, such as *P*. *perniciosus* and *P*. *perfiliewi* [11,12]. During the last decades, the mean annual average of HVL was 99.6 cases/year, with a mean annual incidence of 1.04 cases/100,000 inhabitants [9], indicating an important increase with respect to previous studies. Indeed, the mean incidence rate rose from 12.8 cases in the 1960s and 1970s to 56.8 cases in the 1980s. As of the 1990s, it reached epidemic levels of approximately 100 cases [9,13–15].

A spatial correlation was confirmed between the occurrence of HVL and the high rate of *Leishmania* infection in Tunisian dogs, which are the main reservoir host of *L*. *infantum* [10,14,16]. In addition, previous serological surveys carried out in several regions of Tunisia also confirmed the endemic and progressive nature of CanL and showed that it is expanding to new parts of the country previously presumed to be free of the disease–the central and southern regions [10,14,17–19].

Several factors, including environmental and climatic changes affecting the presence and density of the phlebotomine sand fly vectors, are suspected causes of the epidemiological changes of leishmaniasis in North Africa, particularly in Tunisia [5]. The surveillance of the expansion of canine leishmaniosis and its vector sandflies is essential to understand the epidemiology of the disease and to develop a health control program in Tunisia for both humans and dogs.

The primary objective of the present work is to study the prevalence of canine leishmaniosis by bioclimatic region using serological and molecular techniques to detect *L*. *infantum* infection in dogs. We also aimed to collect sand fly fauna in the sampled dog’s surroundings, to identify the role of these insects in the spread of CanL and contribute to developing a disease control strategy.

## Methods

### Ethics statement

This study was approved by the ethical review committee of the Institute Pasteur of Tunis. Dogs were examined and sampled by veterinary clinicians following procedures of this committee and after obtaining the owner’s consent. Owners presented their dogs for rabies vaccination.

### Study area and data collection

**Fig 1:**
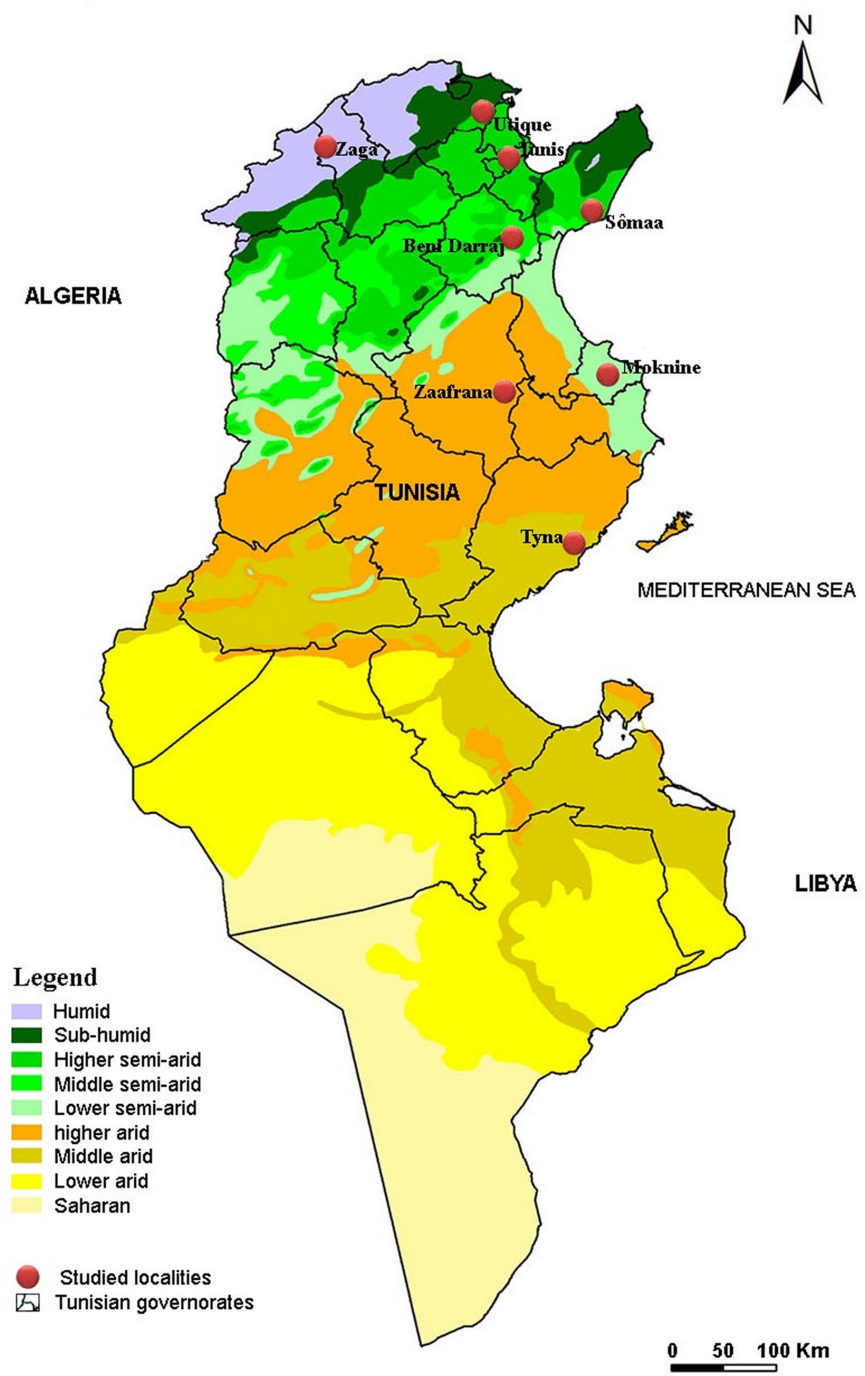
Map of Tunisia showing the studied localities according to bioclimatic zones. The base layers of the map is on this link: https://esdac.jrc.ec.europa.eu/content/carte-bioclimatique-de-la-tunisie-selon-la-classification-demberger-etages-et-variantes.

A cross-sectional study was carried out in 8 localities, representing 8 bioclimatic zones in Tunisia (Table 1 and Fig 1). Mixed-breed dogs were randomly sampled during rabies vaccination campaigns organized by regional veterinary services between June and August 2016. Informed consent was obtained from each owner. Stray dogs were not included in our study as they were difficult to catch. All dogs were subjected to a rapid physical examination for compatible clinical signs with CanL, including skin lesions, exfoliative dermatitis, erosive-ulcerative lesions, lymphadenomegaly, weight loss, and onychogryphosis [20].

**Table 1:** Prevalence rates of *L*.*infantum* in dogs determined by IFAT (1:80) and PCR according to the locality.

Studied locality (Governorate)	Geographic Coordinates	Bioclimatic zones (annual rainfall)	Agricultural landscape and natural vegetation	IFAT positive dogs/ tested dogs seroprevalence % [95%CI]	PCR positive/tested dogs prevalence % [95%CI]
**Zaga (Béja)**	**36°55’20.41" N** **8°59’30.6"E**	Humid (950 mm).	Rural. Mountainous site with oaks, river whose water used to irrigate vegetable crops and tobacco. Cattle breeding	33/46 71.7 [58.7–84.7]	6/44 13.6 [3.5–23.7]
**Utique (Bizerte)**	**37° 6’36.68"N** **10° 1’18.74"E**	Sub-humid (600 mm)	Rural. Hilly, grain cultivation, cattle breeding	18/31 58 [40.7–75.4]	8/31 25.8 [10.4–41.2]
**Tunis**	36°51’52.1"N 10°10’53.2"E	Higher semi-arid (450 mm)	Peri-urban. Small home gardens	14/32 43.7 [26.5–60.9]	9/29 31 [14.2–47.8]
**Sômaa (Nabeul)**	36°32’31.7"N 10°46’50.6"E	Middle Semi-arid (costal) (450 mm)	Rural. Agricultural, primarily citrus trees and market gardening. Very few cattle	26/33 78.8 [64.8–92.7]	9/32 28.1 [12.5–43.7]
**Beni Darraj (Zaghouan)**	36°24’6.53"N 10°15’40.02"E	Middle Semi-arid (continental) (400 mm)	Rural. Flatlands, grain production. Cattle and sheep breeding	22/29 75.8 [60.3–91.4]	7/27 26 [9.4–42.4]
**Moknine (Monastir)**	35°36’13.84"N 10°53’27.40"E	Lower semi-arid (250 mm)	Rural. Coastal, relatively low, olives trees. Poultry	25/50 50 [36.1–63.8]	10/48 20.8 [9.3–32.3]
**Zaafrana (Kairouan)**	35°32’41.74"N 10° 4’27.35"E	Higher arid (250 mm)	Rural. Plains, olive, and other fruit trees. Sheep and poultry farming	44/52 84.6 [74.8–94.4]	12/50 24 [12.16–35.8]
**Thyna (Sfax)**	34°39’29.95"N 10°40’7.18"E	Middle arid (200 mm)	Rural. Lowland, olive trees. Poultry farming	3/44 6.8 [0–14.3]	3/41 7.3 [0–15.3]
**Total**				**185/317** **58.3** [52.9–63.8]	**64/302** **21.2** [16.6–25.8]

A sample of peripheral blood (5 mL) was taken from each dog’s radial vein. Whole blood was collected in tubes with no additives for an indirect fluorescent antibody test (IFAT) and also in tubes with Ethylenediaminetetra-acetic acid (EDTA)-anticoagulant for DNA extraction and PCR analysis. Data for each dog included age (3–8 months, 9–18 months, 18–36 months, more than 36 months), gender, and the sampling site’s bioclimatic zone (Fig 1 and Table 1). According to the owners, no insecticide or other treatment has been used against sandflies.

### Detection of *Leishmania infantum*

#### Serological survey

The seroprevalence of *L*. *infantum* was investigated using a commercially available IFAT. The *Leishmania*-spot IF test (BioMérieux, Marcy l’Etoile, France) was carried out as per the manufacturer’s recommendations. Sera were screened at a single dilution of 1:80 in Phosphate-buffered saline (PBS) and considered positive for anti-*Leishmania* antibodies when two readers observed fluorescence at this dilution. Dog sera and controls (positive and negative) were diluted to 1:80 in PBS. Aliquots of 10 μL were spotted on each circle and slides were incubated for 10 min at 37°C with 95% humidity. Fluorescent staining was performed using a fluorescein-labeled anti-dog gamma globulin (BioMakor, France) diluted at 1:100 and colored with 0.002% Evans blue-PBS solution.

### Molecular survey

#### DNA extraction

DNA was extracted from 200 mL of whole blood from each dog using the DNeasy Blood and Tissue kit (QIAGEN GmbH, Hilden, Germany), as per the manufacturer’s instructions. DNA was eluted on 100 μL and extracts were stored at −20°C until use. Distilled water was included as a negative control for every 10 samples to test for possible contamination. DNA concentration was examined with a NanoDrop ND-1000 spectrophotometer (NanoDrop Technologies, Wilmington, DE).

#### PCR amplification

A set of primers RV1 and RV2 was used to amplify the 145 bp of the conserved region of *L*. *infantum* kinetoplast DNA [21]. These primers are specific to *L*. *infantum*, which belongs to the *L*. *donovani* sensu lato complex. Using a commercially available PCR assay kit, PCR reactions were performed in 25 μL final volume reactions that included 0.5 μM of each primer, 200 μM (each dNTP) (Takara, Japan), 0.75 U Taq DNA Polymerase (TaKaRa Ex Taq, Hilden, Germany), 1× Taq buffer and 4 μL extracted genomic DNA. Amplification was carried out in a thermocycler (Applied Biosystems 2720, Germany) as follows: initial annealing at 94°C for 4 min, 40 cycles of 94°C for 30 s, 59°C for 30 s, and 72°C for 30 s, a final elongation at 72°C for 10 min. To avoid cross-contamination and false-positive reactions, PCRs were performed in a separate room, used plugged tips, and included a negative (water) and a positive control in each run. PCR products were visualized by SYBR Safe DNA Gel Stain (Invitrogen, France), staining after electrophoresis in a 1.5% Agarose gel.

#### Entomological survey

Phlebotomine sand fly sampling was performed in June and July 2016, the main phlebotomine sand fly season [12]. Sandflies were collected using Centers for Disease Control and Prevention miniature light traps (CDC-LT) (John W. Hock Co., Gainesville, FL, U.S.A). In each sampling site, four CDC-LT were suspended proximate to the dog’s habitat and operated for 2 nights between 6:00 pm and 08:00 am. CDC-LT were then transferred to the laboratory and kept in the freezer for 15 min to immobilize collected phlebotomine sandflies. Specimens were examined under a binocular dissecting microscope and identified at the species level according to the morphological characteristics described by Croset et al., [22] and Ghrab et al., [23]. The relative abundance of each species was estimated as the percentage of samples collected per species from the total number of species collected.

#### Statistical analysis

Statistical analysis was carried out with R software [24]. Descriptive methods were used to characterize dogs sampled and the diagnostic test results (IFAT and PCR). Proportions were presented for categorical variables and 95% confidence intervals (CI) were estimated. Chi-square was used to test the associations between possible risk factors and both the presence of the parasite (PCR) and antibodies against *L*. *infantum* (IFAT). When the sample size was small, Fisher’s exact test was used instead. The differences were considered statistically significant with a p-value was ≤0.05. Odds ratios (OR) and the 95% CI were estimated by binary logistic regression. The degree of agreements between IFAT, PCR results, and presence of symptoms were determined by calculating the Kappa (k) values with 95% CI. Kappa was considered statistically significant with a p-value ≤ 0.05.

## Results

A total of 317 dogs were sampled. The sex ratio was 2:1 (214 males: 103 females). The average age of the dogs was 2.8 years (ranging from 5 months to 16 years). Of the dogs that were examined, 41 (12.9%) presented at least two clinical signs compatible with leishmaniosis.

### IFAT test

Among 317 tested dogs by IFAT (single dilution 1:80), 185 (58.3%) presented IgG anti-*L*. *infantum* (Table 2). Only 31 (16.7%) of the seropositive dogs presented clinical signs suggestive of CanL. Seroprevalence rates varied from 6.8% to 84.6% according to bioclimatic zone and between 28% and 66% according to age group (Table 1). Both age and bioclimatic zones were significantly associated with the presence of antibodies against *L*. *infantum* (p<0.05). In female dogs, the seroprevalence rate (61%; 63/103) was higher than in males (57%; 122/214), but this difference was not statistically different (p>0.05) (Table 2).

**Table 2:** Descriptive of CanL prevalence.

Risk Factors	IFAT			PCR		
	Nb positives /Nb tested dogs	Prevalence [95%CI]	OR [95%CI]	Nb positives/ Nb tested dogs	Prevalence [95%CI]	OR [95%CI]
**Sex**						
Female	63/103	0.61 [0.52–0.70]	Reference	23/98	0.20 [0.14–0.25]	Reference
Male	122/214	0.57 [0.5–0.64]	1.188 [0.735–1.919]	41/204	0.23 [0.15–0.32]	0.688 [0.372–1.274]
**Age**						
3–8 months	7/25	0.28 [0.10–0.45]	Reference	1/13	0.7 [0–0.23]	Reference
9–18 months	24/50	0.48 [0.34–0.61]	0.223 [0.087–0.572]	10/42	0.23 [0.10–0.36]	0.193 [0.025–1.487]
>18–36 months	42/74	0.56 [0.45–0.68]	0.470 [0.248–0.891]	19/74	0.25 [0.15–0.35]	1.168 [0.524–2.603]
> 36 months	112/168	0.66 [0.59–0.73]	0.648 [0.371–1.130]	35/173	0.20 [0.14–0.26]	1.356 [0.711–2.583]
**Bioclimatic zones**						
Sub-humid	18/31	0.58 [0.40–0.75]	Reference	8/31	0.25 [0.10–0.4]	Reference
Humid	33/46	0.71 [0.58–0.84]	1.833 [0.702–4.786]	6/44	0.13 [0.35–0.23]	0.454 [0.140–1.475]
Higher semi-arid	14/32	0.43 [0.26–0.60]	0.562 [0.207–1.524]	9/29	0.31 [0.14–0.47]	1.294 [0.420–3.986]
Middle Semi-arid (costal)	26/33	0.78 [0.64–0.92]	2.683 [0.895–8.042]	9/32	0.28 [0.12–0.43]	1.125 [0.369–3.427]
Middle Semi-arid (continental)	22/29	0.75 [0.60–0.91]	2.270 [0.748–6.888]	7/27	0.26 [0.94–0.42]	1.006 [0.310–3.269]
Lower semi-arid	25/50	0.50 [0.36–0.63]	0.722 [0.293–1.783]	10/48	0.20 [0.93–0.32]	0.757 [0.261–2.193]
Higher arid	44/52	0.84 [74.8–0.94]	3.972 [1.408–11.210]	12/50	0.24 [0.12–0.35]	0.908 [0.323–2.552]
Middle arid	3/44	0.60 [0–0.14]	0.053 [0.013–0.208]	3/41	0.73 [0–0.15]	0.227 [0.055–0.943]

Nb: Number; OR: Odds Ratio; CI: Confidence Interval

### PCR test

A total of 302 dogs were tested by PCR, which yielded 64 positives (21.2%) out of which 12 presented CanL clinical signs (10 weight loss; 5 lymphadenomegaly). In 15 dogs, the blood sample was insufficient to be tested by PCR. The PCR *Leishmania* prevalence varied with age group from 7% to 25%; and among bioclimatic zones (7.3% to 31%) (Table 2). Neither age nor bioclimatic zone were significantly associated with a *Leishmania* infection rate detected by PCR (p>0.05) (Table 2).

### IFAT vs PCR and presence of clinical signs

Only 46 samples (15.2%) were positive by PCR an IFAT, among them only 10 dogs showed CanL sings. Of 136 (45%) dogs positive only by IFAT, 19 showed symptoms (Table 3). The calculated Kappa value with a 95% confidence interval was k = 0.09 (0.07, 0.17) for serology and PCR, indicating a low agreement between the results of these methods. Similarly, low agreement was also observed for both tests and presence of symptoms k = 0.03 (0.09, 0.16) for serology and k was non-significant for PCR (p>0.05) (Tables 3 and 4).

**Table 3:** IFAT vs PCR results and compatible clinical signs of CanL.

IFAT	PCR	Nb of dogs	Presence of CanL symptoms
+	+	46	10
-	-	102	4
+	-	136	19
-	+	18	2
**Total**		302	35

**Table 4:** The agreements between IFAT, PCR results and compatible clinical signs of CanL.

	PCR / IFAT	PCR / CanL Symptoms	IFAT / CanL Symptoms
**Expected Agreement**	0.44	0.72	0.42
**Observed Agreement**	0.49	0.75	0.47
**Kappa coefficient**	0.09	0.1	0.09
**95% Confidence interval**	0.007–0.17	0.003–0.21	0.003–0.15
**Z (Kappa); P value**	2.13; p <0.05	2.01; p <0.05	2.90; p <0.05
**Classification**	Minimal	Minimal	Minimal

### Entomological survey

A total of 3,210 phlebotomine sandflies were captured. Of these, 2,654 (83%; 1,574 males, 1,080 females for a ratio of 1.46) specimens were identified as belonging to 16 species of two genera—*Phlebotomus* and *Sergentomyia*. *P*. *perniciosus*, *P*. *papatasi*, and *P*. *perfiliewi* were the dominant species with relative abundances of 34.7%, 25% and 20.4% respectively. Most sandflies were collected in continental medium semi-arid and lower semi-arid areas (Table 5).

**Table 5:** Entomological survey by locality.

Locality Phlebotomus species	Zaga	Utique	Tunis	Sômaa	Beni Darraj	Moknine	Zaafrana	Thyna	Total	Relative abundance
P. alexandri	0	0	0	0	0	13	0	3	16	0.6
P. ariasi	4	0	0	0	0	0	0	0	4	0.2
P. chabaudi	0	0	0	0	0	12	3	6	21	0.8
P. langeroni	0	0	0	0	0	8	0	0	8	0.3
P. longicuspis	5	13	0	30	0	9	11	16	84	3
P. papatasi	54	2	25	23	221	128	120	88	661	25
P. perfiliewi	85	149	96	28	117	37	20	11	543	20.4
P. perniciosus	70	143	126	45	205	209	91	32	921	34.7
P. riouxi	0	0	0	0	0	9	0	0	9	0.3
P. sergenti	0	0	0	0	0	12	0	0	12	0.4
S. antennata	5	0	0	7	0	6	0	1	19	0.7
S. christophersi	0	0	0	0	0	7	8	0	15	0.6
S. clydei	0	0	0	0	0	5	0	0	5	0.2
S. dreyfussi	0	0	0	0	0	14	29	20	63	2.4
S. fallax	0	0	0	0	0	4	21	13	38	1.4
S. minuta parroti	11	1	18	3	115	58	23	6	235	8.85
**Total**	234	308	265	136	658	531	326	196	2654	100

## Discussion

CanL caused by *L*. *infantum* is the most important vector-borne disease in dogs in the Mediterranean region which also raises human health concerns [25–28]. The IFAT is considered the serological method of choice for diagnosing CanL [29–31]; it is useful for assaying specific IgG against *L*. *infantum* in large numbers of dogs. Using IFAT 1:80 in our CanL survey, we found an overall seroprevalence of 58.3% across the 8 localities in 8 different bioclimatic zones (ranging from 6.8% to 84.6%). This overall seroprevalence is higher than those previously reported in Eastern-Central and Central Tunisia 6% and 26.7% respectively [10,17]. Similarly, in the north of Tunisia Diouani et al., [18] showed seroprevalence rates of 18% and 22% in 1994 and 1995 respectively. In neighboring Algeria and Morocco, seroprevalence rates varied by region and by test with Algeria at 36% [28] and Morocco at 18.7%) [32].

This seroprevalence is also in the range of rates in highly endemic regions of several Mediterranean countries such as Spain, Italy, and France, where the mean reported seroprevalence varied between 65% and 98% [25,33]. Seroprevalence in our study, varied significantly among bioclimatic zones ranging from 84.6% to 6.8%. Zoghlami et al., [10] reported an overall CanL seroprevalence of 26.7% with a maximum of 52.7% in central Tunisia. Similar variations in prevalence were recorded in Morocco, Algeria, and Iran [28,32,34,35].

The easily-performed IFAT remains the reference serological method for the detection of antibodies against *Leishmania* in dogs [31]. Like any diagnostic technique, it comes with limitations, such as the significantly lower sensitivity for identifying asymptomatic dogs as compared with tests like ELISA [36,37].

Nevertheless, in endemic areas, IFAT 1:80 positive dogs does not confirm the infection, as it could only indicate exposure to the parasite [38]. To confirm the infected dog, an end-point serial serum dilution should be obtained to determine the level of the antibodies [39]. Indeed, dogs with higher IFAT titer are known to display more clinical signs of leishmaniosis. Many studies have found a positive correlation between anti-*Leishmania* antibodies and clinical manifestations [40,41]. IFAT may also lead to false-positive results [42] from cross reactivity with antibodies against other infectious agents such as *Ehrlichia*, *Babesia*, *Trypanosoma* [43–46]. In addition, cross reactions can be observed with *Leishmania major* and *L*. *tropica* (*L*. *killicki*) which are endemic in Central and Southern Tunisia [47], and may also infect dogs [48].

To complement the serological test, we used a specific *Leishmania* PCR analysis. Among 302 tested dogs by PCR, only 21% were positive, a prevalence rate lower than that obtained by IFAT but similar to other findings [49]. The low agreement between PCR and IFAT was not surprising. Previous studies have reported conflicting results between PCR and IFAT [50]. In dogs from endemic area in Tunisia, Chargui et al., [51] showed that PCR was more sensitive than IFAT. Indeed, PCR is very sensitive and specific when multicopy DNA sequences are targeted. On another side, there is no consensus among laboratories on the IFAT cut-off titer, which can vary from 1:40 to 1:320; those could explain the variation in seroprevalences [52].

Dogs that are PCR positive but seronegative might have been recently infected, not having detectable antibodies since seroconversion can take several months [53]. Indeed, some dogs can remain antibody-negative indefinitely [26,27]. In fact, seropositive dogs with clinical signs should have a positive PCR, as the parasite load in tissues is higher. So, negative PCR results could be attributed to the DNA extraction protocol, the primers used and the used biological samples [54,55]. Indeed, higher sensitivity values were found for PCR dot blot tests performed on lymph node aspirates than for tests with blood samples [54]. Furthermore, seropositive dogs testing negative by PCR (n = 136) might have a strong, persistent humoral response (IgG) [56,57].

PCR on blood, which is less sensitive when compared to lymph nodes and bone marrow, cannot replace the “Gold Standard test” for a large epidemiological study [44,45]. Less invasive methods such as swab rubbing of the conjunctiva and oral mucosa or snout, did however provide samples with higher parasite loads than blood, and could be used to improve sensitivity of molecular techniques such as PCR, which can be used as complement tests for the diagnosis of Leishmaniosis [37].

Only 16.7% of the seropositive dogs presented clinical signs possibly linked to leishmaniosis. This supports a study in northern Tunisia reporting similar results [18]. It is more likely that naturally selected local dog populations show no symptoms of infection [58], as several studies in CanL-endemic areas have reported that approximately 80% of infected local dogs are asymptomatic [49,59,60] and can remain so for a prolonged period of time [34,61,62]. Such asymptomatic dogs may play an important role in the transmission cycle of visceral leishmaniasis [63] as they can still transmit *Leishmania* to sandflies [63,64].

Seroprevalence did not differ by gender, as previously described in Tunisia [18] and elsewhere [25,61,65,66]. Seropositivity does increase significantly with age; older dogs have a higher risk of exposure to infected phlebotomine sandflies [25,34,61,65]. In puppies (less than 8 months) seropositivity (28%) is higher comparing to the PCR rate (7%). This could be attributed to the vertical transmission of *Leishmania* parasite as it was previously shown. This transplacental transmission of *Leishmania* may be considered a factor in the increase of seroprevalence [67–69].

All Phlebotomine identified in this study were previously recorded, which indicate that no new *Phlebotomus* species have been detected in this investigation. The number of night trap samples surveyed, during our investigation, do not allow definitive statement about sand fly fauna. However, they do provide information about sandflies living in proximity of dogs. Phlebotomine vectors were collected in July, the normal season for sand fly activity in Tunisia [22,23]. Previous studies strongly suspected *P*. *longicuspis*, *P*. *langeroni* and *P*. *ariasi* were the main vectors of *L*. *infantum* in North Africa [11,70,71]. Interestingly, this study caught very few sandflies of these species compared to *P*. *perniciosus* Newstead, 1911 and *P*. *perfiliewi* Parrot, 1939 which were found in proximity to dogs at all localities of the study and represented 55% of all captured specimens. This finding matched other studies that considered these two species to be the main vectors of *L*. *infantum* in the Mediterranean basin [11], especially in North African countries [10,12,71]. Both *P*. *perniciosus* and *P*. *perfiliewi* are opportunistic feeders [72,73] and the principal vector of the viscerotropic *L*. *infantum* zymodeme MON1, the etiologic agent for both CanL and HVL in Tunisia [74,75]. *P*. *papatasi*, was quite abundant (25%) in our Phlebotomine collection. This species, known as the vector of *L*. *major* and the agent of cutaneous leishmaniasis in the central and southern Tunisia, can bite dogs and transmit *L*. *major* [48].

This study confirms that CanL is gaining ground in Tunisia. The parasite first found to be endemic in the sub-humid and upper semi-arid zones of northern Tunisia [76–79] has slowly expanded southward [9,10,14,17,80,81]. Our study showed a clear increase in CanL seroprevalence, particularly in the arid zone (49% vs 1.6%) [82]. Boussaa et al., [32] reported a similar south ward geographical expansion in Morocco where a CanL seroprevalence of 82% was found in southern regions that had been considered disease-free merely a few years ago.

Changes in agricultural practices and land use in the last few decades may contribute to this growth in prevalence in Tunisia’s central and southern regions [83]. Indeed, ecological changes can affect the abundance of some wildlife species which can be a reservoir of *Leishmania*. Indeed, in some regions of Tunisia, Chemkhi et al., [84] have observed an abundance of hedgehogs and demonstrated they are infected by *L*. *infantum*. In addition, ecological changes, subsequent to the construction of a dam in central Tunisia, were at the origin of the abundance of rodents (*Meriones shawi* and *Psammomys obesus*) reservoirs of *L*. *major* that caused an epidemic zoonotic cutaneous leishmaniasis in local human population [5]. The migratory habits of mammal reservoirs may contribute to spreading out the disease. Worldwide, several wild animals have been reported to act as reservoirs in the transmission cycle of leishmaniasis, including in North Africa [85,86]. The proliferation of wild animals and rodents, in particular, offers a suitable habitat and blood sources to sandflies, which contribute to increasing vector abundance [87]. Studying the role of these animals is highly complex and requires multidisciplinary expertise a One Health approach.

Recent increases in livestock farming, particularly of sheep [88], poultry, and cattle may have created a propitious context for the exponential growth of phlebotomine species [83,89,90]. In fact, the development of animal husbandry, especially in Tunisia’s semi-arid and arid regions, has produced enormous quantities of poultry feces and livestock manure that are favorable breeding sites for the development of phlebotomine sand fly larvae. Farmers also use manure and poultry feces to enrich the soil. The majority of farmers in arid areas irrigate with groundwater pumped from wells. This change in land use generates moisture in a soil enriched with organic matter that makes it more favorable for phlebotomine larval development, particularly *P*. *perniciosus* and *P*. *perfiliewi*. Indeed, the larvae of these phlebotomines colonize domestic environments and are adapted to moist soil enriched with organic matter (livestock excrement) in which females have most probably laid eggs [91–93]. Climate change, including global warming, are proven influences on vector abundance and therefore on CanL prevalence [94,95]. In central Tunisia (Kairouan), average temperatures oscillating between a 20°C minimum and 38°C maximum from June-September (the active period of phlebotomines) are quite favorable for the development of *Leishmania* in the vector. The Intergovernmental Panel on Climate Change (IPCC) projects a likely rise in global mean temperatures of 1.5°C between 2030 and 2052 [96]; the Mediterranean region is considered one of the most sensitive areas for such higher temperatures.

Ecological, agronomic, social, and climatic factors have all contributed to the expansion of zoonotic visceral leishmaniasis in dogs and in humans in Tunisia and elsewhere. In Tunisia, agricultural practices such as breeding activities are the primary factor in favoring the establishment and proliferation of phlebotomine sandflies and must, therefore, be considered in any leishmaniasis control program. Consequently, and in view of our results and field observations, manure should be buried in the ground and plowed frequently as an additional prevention for zoonotic leismaniasis in endemic areas. In addition, (i) stray and owned dogs should be monitored, and health and preventive methods should be put in place (e.g. sterilization, insecticide-impregnated dog collars, vaccine), (ii) Sand fly population should be controlled with insecticides in nearby animal shelters and potential phlebotomine sand fly habitats [97]. Adopting all of these strategies together has been suggested. We note that euthanizing infected dogs as a control method is unacceptable for ethical and social reasons and was therefore abandoned in Tunisia.

## Conclusion

In conclusion, this epidemiological survey highlighted that CanL infection in dog populations is prevalent in all bioclimatic areas in Tunisia and confirms the ongoing spread of the infection of dogs in arid zones. This expansion of infection in the dog population may well result from ecological, agronomic, social and climatic factors affecting the presence and density of phlebotomine vectors and wild and domestic animals but further investigations in CanL in arid regions are necessary to identify the factors implicated in the rapid spread and evolution of this disease to develop better prevention measures for humans and animals alike.
